# Global research trends and hotspots analysis of hallux valgus: A bibliometric analysis from 2004 to 2021

**DOI:** 10.3389/fsurg.2023.1093000

**Published:** 2023-03-07

**Authors:** Shulong Wang, Ping Deng, Xiaojie Sun, Jinglu Han, Shanshan Yang, Zhaojun Chen

**Affiliations:** ^1^ School of Graduates, Beijing University of Chinese Medicine, Beijing, China; ^2^ Department of Hand and Foot Surgery, Beijing University of Chinese Medicine Third Affiliated Hospital, Beijing, China

**Keywords:** bibliometric, hallux valgus, hotspots, trend, visualization

## Abstract

**Background:**

Hallux valgus (HV) is a common foot and ankle surgery disease. The correction of HV deformity relies on a highly challenging surgical treatment. Thus, widely adopted evidence-based clinical guidelines are still needed to guide the selection of the most appropriate interventions. Recently, the study of HV has been growing and scholars are increasingly paying particular attention to this area. However, bibliometric literature remains lacking. Therefore, this study aims to reveal the hotspots and future research trends in HV *via* bibliometric analysis to fill this knowledge gap.

**Methods:**

Literature related to HV from 2004 to 2021 was retrieved from the Science Citation Index Expanded (SCI-expanded) of the Web of Science Core Collection (WoSCC). Quantitative and qualitative analyses of scientific data are performed using software such as CiteSpace, R-bibliometrix, and VOSviewer.

**Results:**

A total of 1,904 records were identified for analysis. The United States had the most number of published articles and total citations. Thus, the United States has made an essential contribution to the field of HV. Meanwhile, La Trobe University in Australia was the most productive institution. Menz HB and *Foot & Ankle International* were the most influential authors and the most popular journals among researchers, respectively. In addition, “older people,” “chevron osteotomy,” “Lapidus,” and “hallux rigidus” have always been the hotspots of attention. Changes and developments in the surgery of HV have gained researchers' interest. Future research trends are more focused on “radiographic measurement,” “recurrence,” “outcome,” “rotation,” “pronation,” and “minimally invasive surgery.” Thus, focusing on these subject directions can facilitate academic progress and provide the possibility of better treatments for HV.

**Conclusion:**

This study summarizes the hotspots and trends in the field of HV from 2004 to 2021, which will provide researchers with an updated view of essential information and somehow guide future research.

## Introduction

Hallux valgus (HV) is a common foot and ankle surgery disease, often with symmetrical onset (1, 2). The main clinical manifestations of HV are the lateral deviation and displacement of the hallux at the first metatarsophalangeal joint accompanied by pain and deformity (3). However, the pathogenesis of HV remains unclear, which may be closely related to abnormal bone structure, soft tissue imbalance, shoe-wearing habits, heredity, and other related factors (4, 5).

The incidence of HV was approximately 23% in adults aged 18–65 years and 35.7% in those over 65 years old (6). With progressive subluxation of the first metatarsophalangeal (MTP) joint, foot function is disrupted, thereby resulting in postural instability and an increased risk of falls in older adults (7). Orthopedic surgery is the primary treatment modality for HV. HV correction surgery is one of the 10 most common foot and ankle surgeries in the United States (8). More than 150 surgical procedures have been used to treat HV, but most have now been eliminated considering that surgical modalities are constantly being updated. Therefore, percutaneous or minimally invasive procedures are gaining popularity, but have been controversial compared to traditional open osteopathic techniques (9–11). Despite the high prevalence of HV and the associated quality of life impairments, no widely adopted evidence-based clinical guidelines can guide the selection of the most appropriate surgical approach (12). While some systematic reviews and meta-analyses focus on a specific aspect of HV, the proliferation of scientific publications has made it increasingly difficult for researchers to keep track of recent discoveries and capture the latest hotspots and trends, even within their fields of expertise.

The bibliometric analysis provides a complete overview of a huge body of study literature (13). It expands previously unappreciated insights by providing quantitative and objective identification of historical and current research subjects (14, 15). Bibliometric methods have been increasingly used in medicine over the past few years. However, only few bibliometric studies in the field of HV research have been conducted. Ferreira GF's article focused on a general knowledge framework (16). Accompanied by continuous improvements in bibliometric software, the number and methodologies of the included literature, and the content of the analysis, sophisticated visualization tools are essential in HV applications, especially in research direction diversification to search for global research trends and hotspots. Therefore, this paper primarily aims to: (I) analyze the general knowledge structure and major contributors (country, institution, author, and journal); (II) clarify the research topic, research hotspots, and research directions; and (III) anticipate future research trends.

## Methods

### Data source and search strategy

Web of Science Core Collection (WoSCC) is the world's largest comprehensive academic information resource covering the broadest range of disciplines (17, 18). Hence, it is widely used in the scientific fields as one of the most regularly used databases for bibliometrics (19). This study used the Science Citation Index Expanded (SCI-expanded) of the WoSCC despite the availability of other scientific databases, such as Scopus, PubMed, or EMBASE. However, different databases include different methods of forming literature, export formats of documents, and so on, and thus it may not be appropriate to combine multiple data from other databases (20). First, this study used the subject term “hallux valgus” to search articles and reviews published from 2004 to 2021. The following were the search terms: TS = (“hallux valgus” OR “bunion*” OR “hallux abduct*” OR “metatarsus primus varus”). However, only the English language was accepted. All retrievals were completed and downloaded on September 20, 2022.

### Inclusion and exclusion criteria

**Figure 1:**
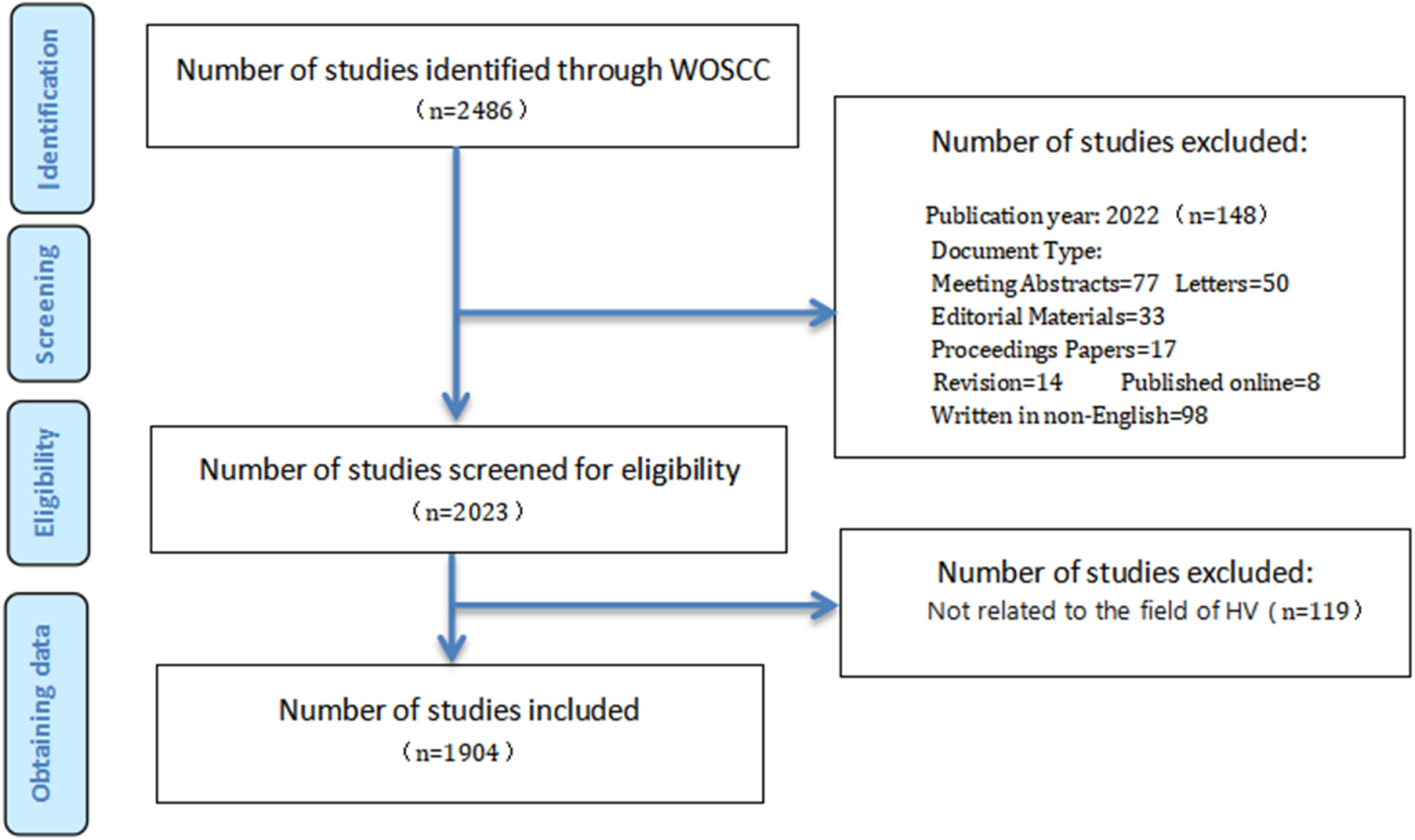
Retrieval strategy and number of publications.

Following the above search strategy, retrieved datasets were exported as “full record and cited references” for further analysis. The titles, abstracts, and keywords of the datasets were read one by one. Inclusion criteria were HV-related articles and reviews (e.g., epidemiological investigations, clinical treatment, recurrence, outcome, etc.) containing all study types. Meanwhile, exclusion criteria were articles and reviews unrelated to HV studies (including disease type, research purposes, interventions, outcome indexes, etc.). The data collection and entry are independently determined by two authors (WS and PD). The disagreement was a consensus reached by the third author (XS). After pre-processing, a total of 1,904 papers in the field of HV were obtained, including 1,771 articles and 133 reviews. The downloaded information was saved in text format. Informed consent was not necessary because these were secondary data with no personal information. The retrieval process was illustrated in Figure 1.

### Data analysis

A suite of tools for sociometric quantitative research is provided by the Bibliometrix R package. Massimo Aria created Biblioshiny for the secondary development of the Bibliometrix-based Shiny package in R (21). It encapsulates the core code of Bibliometrix and generates a web-based framework for online data analysis. Herein, users can use the interactive web interface to perform relevant scientific measurement and visual analysis work, which reduces the user usage threshold and information input intensity to a certain extent (22). In addition, VOSviewer and CiteSpace were widely used for bibliometric analysis.

Bibliometric analysis was primarily performed using three specific programs from Rstudio's Bibliometrix R package (version 2022.03.10, RStudio team, Boston, MA, United States). This software allows the construction and visualization of bibliometric networks to facilitate research understanding. Particularly, the distribution of each component analyzed in the bibliometric analysis is evaluated by a software package that applies machine learning. Given this, the following variables were used: annual scientific production, most relevant sources, source dynamics (occurrences cumulate, the number was set to 10), source local impact by H-index or total citations (number of sources was set to 10), most relevant authors, top authors' production over time (number of authors was set to 10), author local impact, most relevant affiliations, collaboration network by affiliations, country scientific production, collaboration network by countries, corresponding authors' country, historical direct citation network (number set to 20 nodes), multiple correspondence analysis of high-frequency keywords, tree dendrogram of hierarchical cluster analysis of keywords, and thematic map.

CiteSpace (version 5.7R5W, Drexel University, Philadelphia, PA, United States) was utilized to analyze the following: (1) a dual-map overlay of journals contributed to publications; (2) the top 20 co-citation references with the most citation bursts; (3) timeline distribution of keywords cluster analysis; and (4) the top 20 keywords with the most citation bursts (23). CiteSpace parameters are defined as follows: period (2004–2021), years per slice (1), selection criteria (top 20), and pruning (minimum spanning tree, pruning sliced networks).

Moreover, VOSviewer (version 1.6.17, Leiden University Science and Technology Research Center, The Netherlands) was primarily applied to conduct visualization networks including author co-authorship analysis, cluster visualization of the journal co-citation analysis, and the overlay visualization map of the keywords co-occurrence analysis (24). Nodes indicate authors, journals, keywords, and so on, and the size of nodes and the lines between nodes represent the citation or the number of publications and the interrelationship between nodes (co-authors, co-citation, or co-occurrence), respectively.

## Results

### Annual growth trends in publications

**Figure 2:**
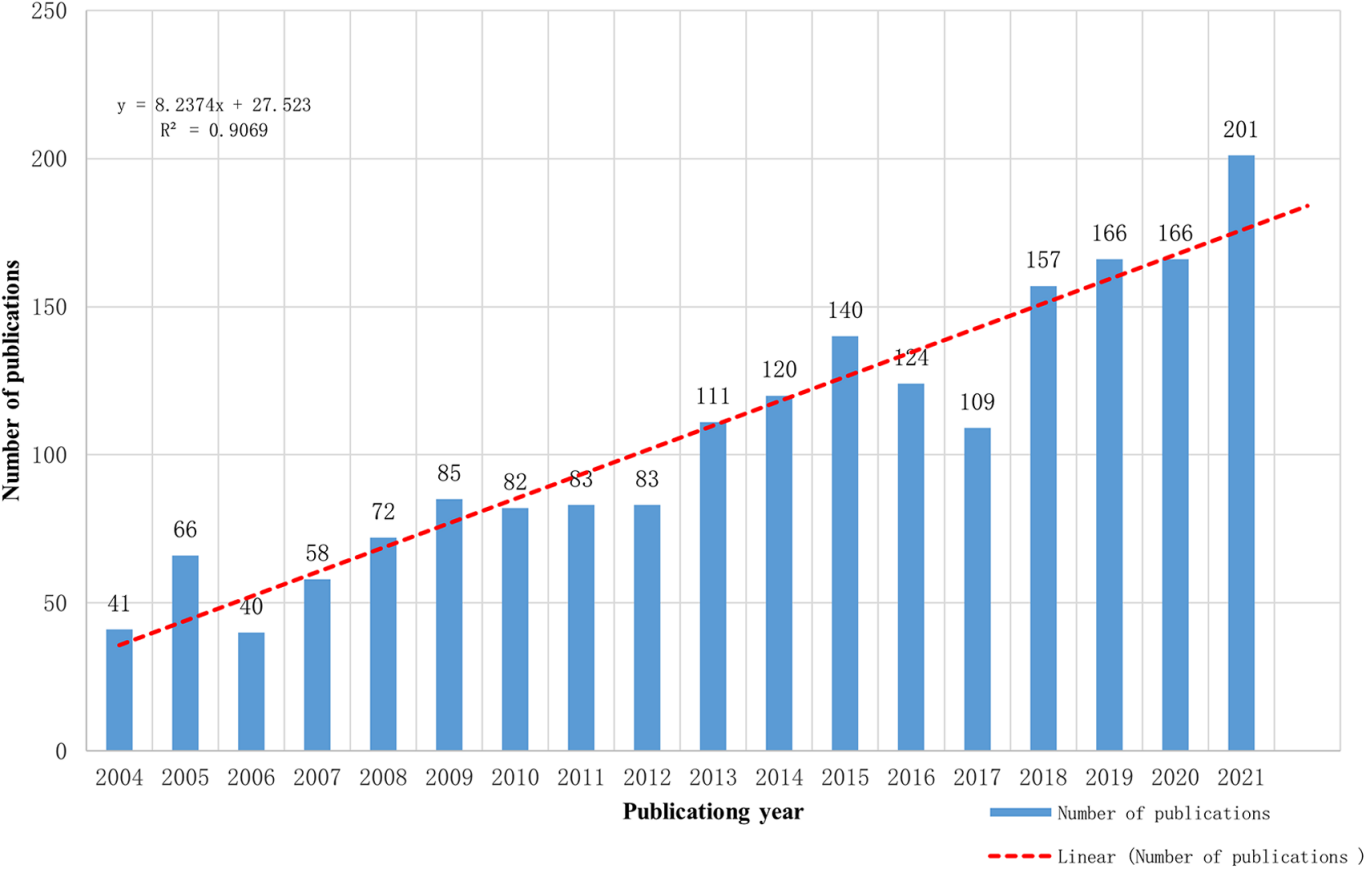
A linear model fitting of publication growth in HV.

Microsoft Office Excel 2019 was used to process the data and constructed a linear model to predict the number of papers published in 2022. Figure 2 showed that the number of papers published in the HV field increased in volatility from 2004 to 2021. An increase was observed with scientific publications growing at an average rate of 34.79%. The linear model of time prediction was established by fitting the formula. A statistically significant relationship was observed between the year and the number of papers (*R*^2 ^= 0.9069) with a good fit. The number of publications in HV would reach approximately 184 in 2022 based on the fitted curve.

### Analysis of major countries/regions

**Figure 3:**
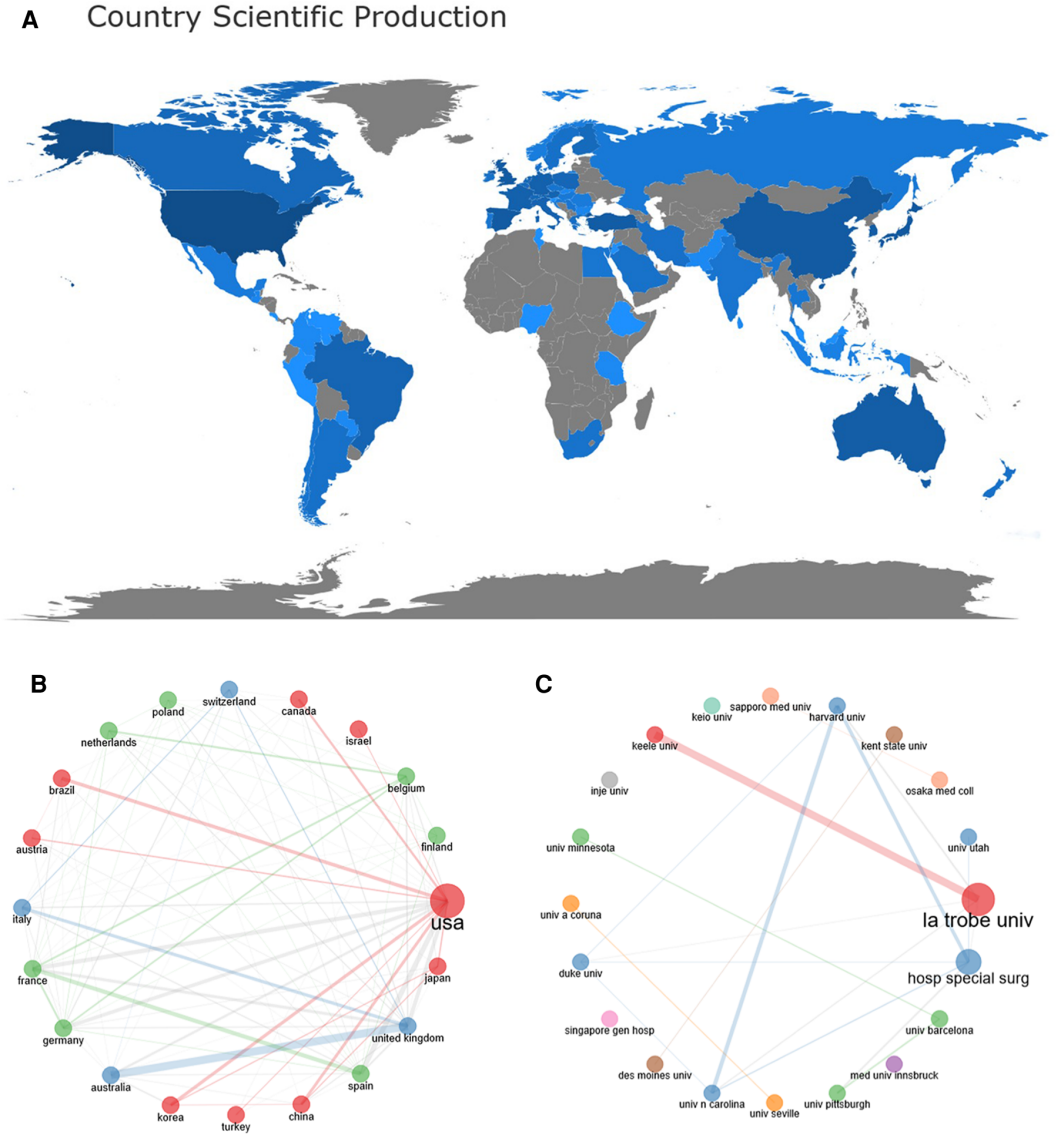
(**A**) distribution of publications from different countries/regions. The color intensity is proportional to the number of documents. Higher blue intensity refers to the greater number of documents, and grey means no scientific output. (**B**) Countries’ collaboration network. There are 20 nodes in the picture. Each node represents a different country and the diameter of each node represents the strength of collaboration between the country and other countries, the line represents the collaboration network or path between each country, and the thickness of the line represents the strength of collaboration. (**C**) Institutions’ collaboration networks in HV.

The distribution features of the significant research countries/regions indicate the influence of countries/regions in the field of HV and create conditions for ongoing growth. The different shades of color shown in Figure 3A represent the number of papers published in each country/region. The top 20 countries or regions in terms of the total publications are primarily from North America, Europe, and East Asia.

The Bibliometrix and Biblioshiny software packages were used to screen for cooperation between countries, as shown in Figure 3B. When the number of nodes was set to 20 and the number of cooperation was more than one, the thicker the border between the two labels, the stronger the cooperation intensity between countries, and vice versa. A country's research quality can be reflected by total citations and average total citations (25). The United States was at the core of international collaboration and has the most published articles and total citations. Thus, the United States has made an essential contribution to the field of HV. Extensive collaboration has been established between countries in North America and Europe. As seen from Table 1, Australia had a relatively lower total number of published articles but a higher average of article citations, which may be related to Australia's La Trobe University. La Trobe University may play an essential role in enhancing national academic influence. While China and Turkey top the list of total published articles, the country's average article citations remain in a low position. The quantity and quality are not yet proportional.

**Table 1:** The top 20 most productive countries and institutions in the field of HV.

Position	Country	NP	Total Citations	Average Article Citations	Institutions	NP
1	USA	527	8,427	15.09	La Trobe Univ	76
2	UK	150	2,735	18.23	Hosp Special Surg	50
3	Japan	131	1,469	11.21	Univ Barcelona	33
4	China	123	1,121	9.11	Med Univ Innsbruck	29
5	Korea	105	1,231	11.72	Univ Pittsburgh	26
6	Turkey	97	517	5.33	Univ Seville	26
7	Spain	94	1,079	11.48	Univ N Carolina	25
8	Australia	81	2,838	35.04	Des Moines Univ	23
9	Germany	53	1,209	22.81	Singapore Gen Hosp	21
10	Italy	52	813	15.63	Duke Univ	20
11	Austria	48	799	16.65	Univ A Coruna	20
12	France	44	522	11.86	Univ Minnesota	19
13	Switzerland	41	402	9.80	Inje Univ	18
14	Netherlands	32	681	21.28	Keele Univ	18
15	Poland	32	149	4.66	Keio Univ	17
16	Brazil	30	272	9.07	Sapporo Med Univ	17
17	Singapore	28	363	12.96	Harvard Univ	16
18	Belgium	22	261	11.86	Kent State Univ	16
19	Canada	17	177	10.41	Osaka Med Coll	16
20	Israel	17	265	15.59	Univ Utah	16

NP, Number of Publications.

### Analysis of major research institutions

The study included a total of 2,025 institutions that made relevant contributions. In terms of number of published papers, Table 1 listed the top 20 institutions, with La Trobe University, Hospital for Special Surgery, University of Barcelona, Innsbruck Medical University, and the University of Pittsburgh being the top five. In terms of institutional cooperation, each node and the diameter of each node represent different institutions and strengths of collaboration between that institution with others, meanwhile, the line and the thickness of the line indicate the network or path of cooperation between each institution and the strength of collaboration, respectively. The number of nodes was set to 20, and 20 institutions were analyzed, as shown in Figure 3C. La Trobe University had the highest network of collaborations, followed by the Hospital for Special Surgery. La Trobe University in Australia and Keele University in the UK have established stable cooperation. However, other institutions, such as Inje University, have less cooperation. Thus, focusing on highly productive research institutions and collaborations can help researchers stay abreast of developments and frontiers in their discipline.

### Analysis of main researchers

**Figure 4:**
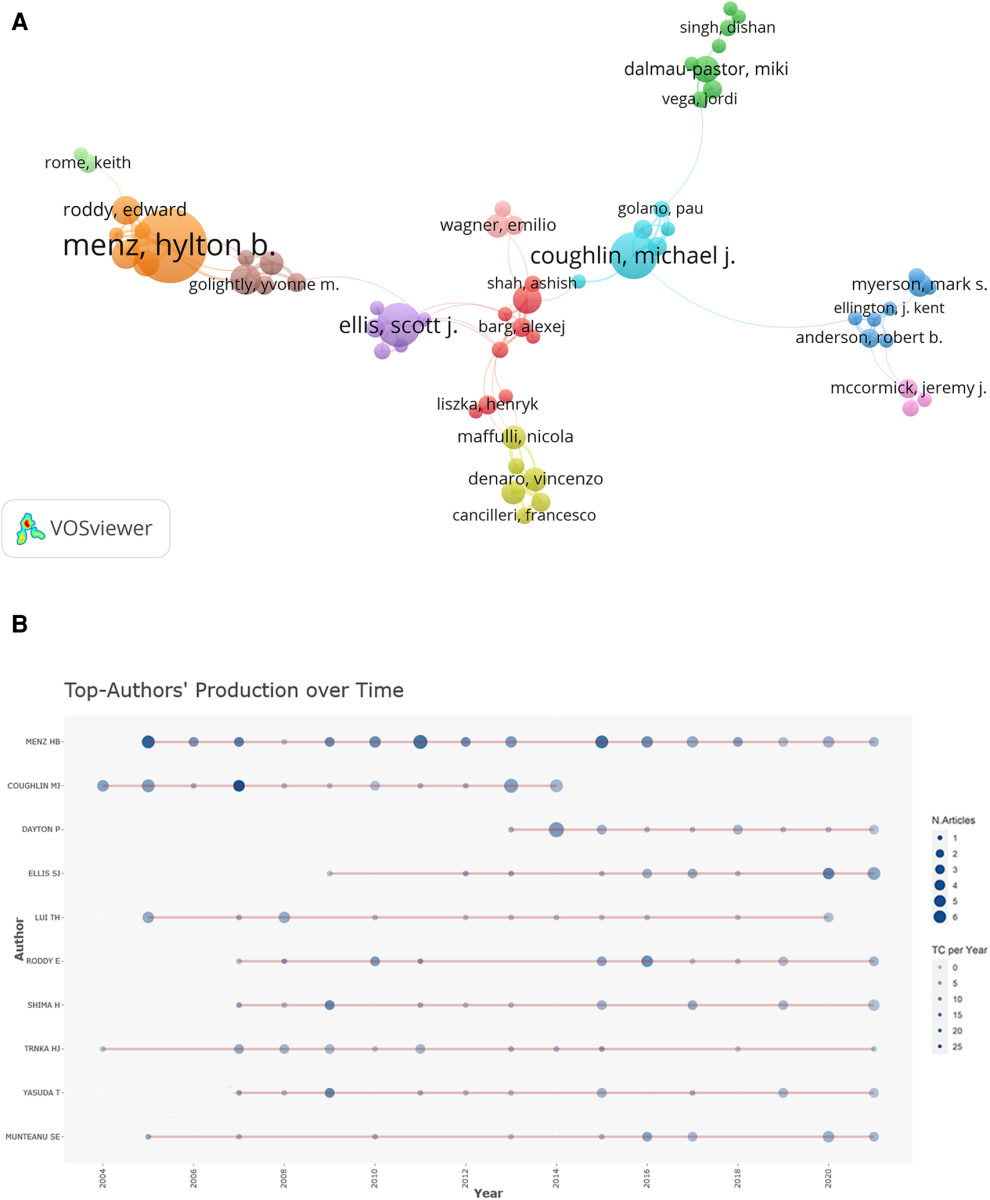
(**A**) network visualization of authors co–authorship in HV. (**B**) Top 10 authors’ productions over time in HV. The circle size shows the number of papers, and the color from light to dark indicates the total citations per year.

Co-author analysis is commonly used to establish a similar relationship between individuals or groups based on the number of co-author publications (26). Figure 4A showed a network map and overlay visualization of 176 co-authors who have published more than five articles. Menz HB, Coughlin MJ, and Ellis SJ were at the center of the collaboration clusters. Table 2 displayed the ranks and they landed in the top three in terms of total number of publications, with 43, 26, and 18 publications, respectively. The H-index and total citations can be used to analyze the quality of current papers and anticipate the performance of future authors, as well as estimate the importance and broad influence of authors' cumulative research contributions (27–29). Menz HB, Coughlin MJ, and Roddy E were the three most influential authors, they are from Australia, the United States, and the United Kingdom, respectively. In HV, the productions of the top 10 authors over time were shown in Figure 4B. The node of Menz HB's most frequently cited paper appeared in 2005 (indicated by the darkest blue color in the circle) and continued to publish in 2021.

**Table 2:** The top 10 authors for volume of publications.

Author	NP	H-index	Total Citations	Institutions	Country
Menz HB	43	24	1,961	La Trobe Univ	Australia
Coughlin MJ	26	17	1,040	Univ Barcelona	Spain
Ellis SJ	18	9	305	Hosp Special Surg	USA
Dayton P	16	9	226	FOOT AND ANKLE CTR IOWA	USA
Lui TH	16	9	312	N DIST HOSP	China
Roddy E	16	10	541	Keele Univ	UK
Trnka HJ	16	11	399	ORTHOPAED HOSP SPEISING	Austria
Shima H	15	10	466	OSAKA MED COLL	Japan
Yasuda T	15	10	478	OSAKA MED COLL	Japan
Koo K	14	6	162	KOREA ADV INST SCI AND TECHNOL	Korea

NP, Number of Publications.

### Analysis of major research journals

**Figure 5:**
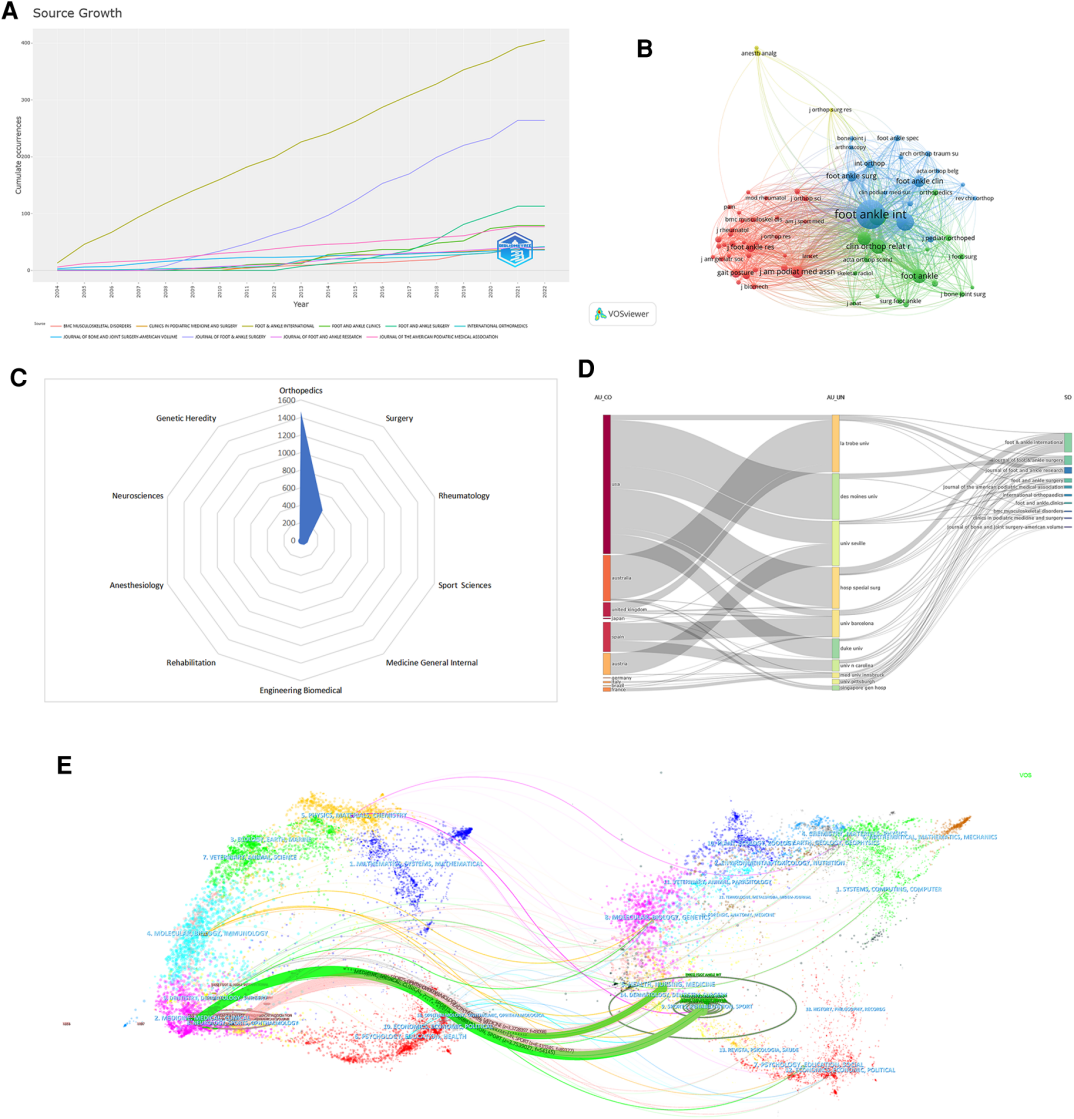
(**A**) cumulative number of publications in the top 10 journals in HV. (**B**) Cluster visualization of the journal co-citation analysis. (**C**) The top 10 research categories ranked by the number of publications. (**D**) The three- field plot showing the knowledge flow (country, institution, and journal). (**E**) The dual-map overlay of journals contributed to publications in HV from 2004 to 2021.

A total of 303 academic journals published papers related to HV research were found within the analyzed time frame. Table 3 listed the names of the top 10 journals by the number of papers published in the professional journals. *Foot & Ankle International* has the highest number of publications (*n* = 405), followed by the *Journal of Foot & Ankle Surgery* (*n* = 264), *Foot and Ankle Surgery* (*n* = 113), *Foot and Ankle Clinics* (*n* = 79), and *Journal of the American Podiatric Medical Association* (*n* = 77). The top 10 journals by total number of publications were primarily from the United Kingdom, the United States, and Germany. The annual changes in the number of papers published in the top 10 journals are shown in Figure 5A. The cumulative number of papers published was 1,130, accounting for about 59.35% of all documents. Therefore, the more indicators used to evaluate a journal, the more objective and comprehensive it will be. *Foot & Ankle International* was the journal with the highest total citations and H-index (total citations = 7,473, H-index = 41). It suggested that this journal has more academic influence and attention from researchers in the field of HV. The results of the visualization analysis of journal co-citation were shown in Figure 5B and the top 10 research categories ranked by the number of publications were shown in Figure 5C. The three major research categories of most interest in this field were orthopedics, surgery, and rheumatology.

**Table 3:** Distribution of the top 10 productive journals in HV.

Rank	Journals	Articles	H-index	Total Citations	Country
1	Foot & Ankle International	405	41	7,473	USA
2	Journal of Foot & Ankle Surgery	264	23	2,262	UK
3	Foot and Ankle Surgery	113	15	802	UK
4	Foot and Ankle Clinics	79	16	750	UK
5	Journal of the American Podiatric Medical Association	77	14	476	USA
6	International Orthopaedics	42	16	634	Germany
7	Journal of Foot and Ankle Research	40	17	972	UK
8	BMC Musculoskeletal Disorders	37	11	433	UK
9	Clinics in Podiatric Medicine and Surgery	37	7	173	UK

The double-map overlay of journals reflects the citation relationships between disciplines, thereby highlighting paradigm shifts in research across disciplines (30). In Figure 5E, the left and right sides represent the citing journals and the reference journals (cited journals), respectively. Overall, there were four colored paths, including two green trails and two pink trails.

Different colored rectangles represent significant features in the figure. The height of a rectangle in a three-domain diagram depends on the ratio or value of the sum of the relationships that arise between the rectangle's components (country, institution, and journal) (31, 32). Finally, a three-field plot was drawn, where each column is limited to 10 variables. The most productive countries collaborating with top scientific institutions and publishing academic results in international journals are shown in Figure 5D.

### Historical cited papers of HV

The historical direct citation network for HV was investigated to explore the changes in the relevant research content over time. The Bibliometrix installation package in R Studio was used to perform the historical citation visualization analysis and selected 20 nodes to find essential publications and classical studies on the subject. CiteScore was released in December 2016 and is the most recent indicator. This study examines the methods and substance of classical literature using LCS and GCS. The reference scores in the downloaded dissertation dataset and WoSCC database are referred to as LCS and GSC, respectively (33, 34). These two indicators reflect the contribution of an article to the research field from a different perspective, with a higher score indicating that the researcher values the article more.

**Figure 6:**
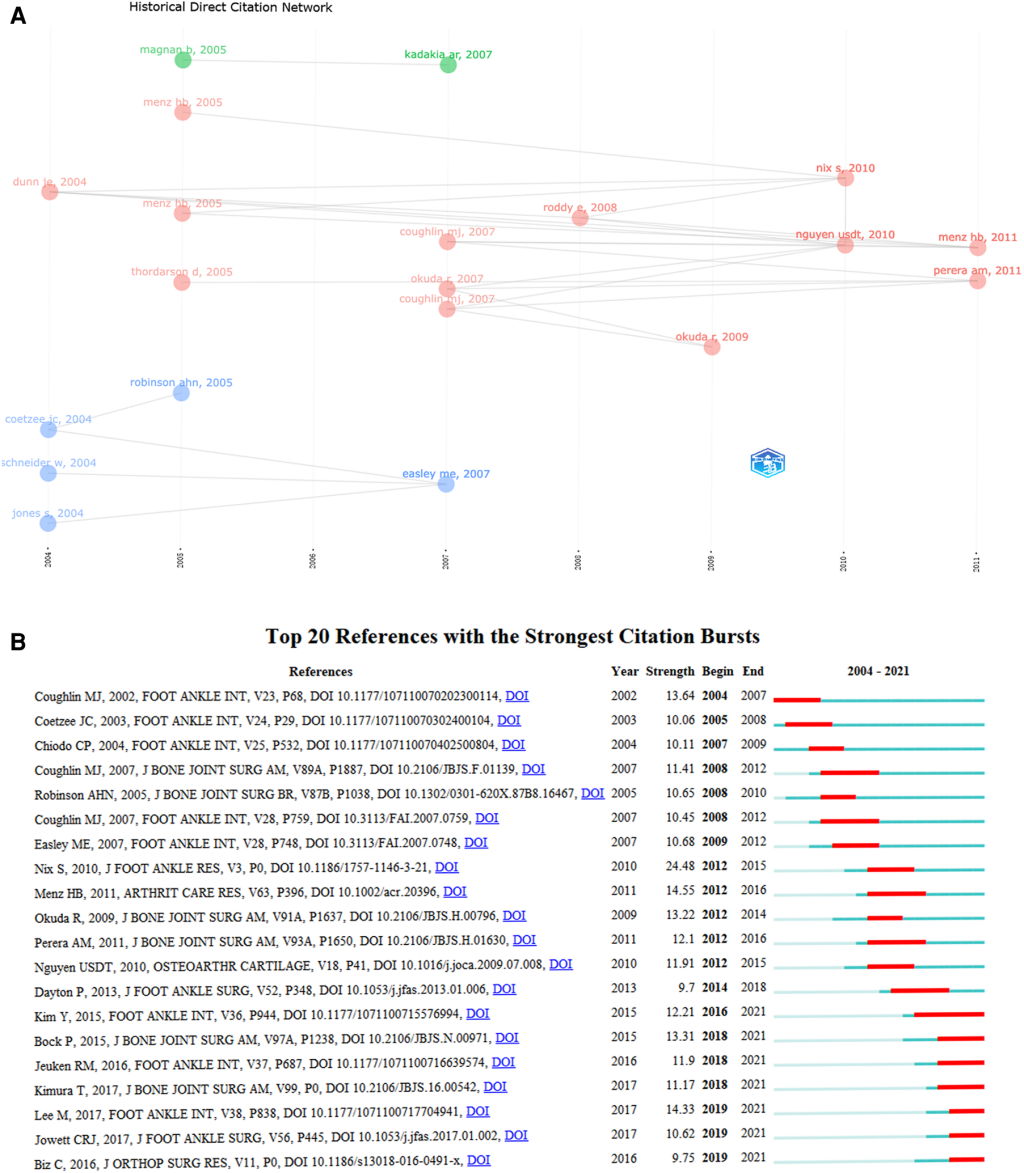
(**A**) historical direct citation network of HV. (**B**) The top 20 co-citation references with the most citation bursts.

As seen in Figure 6A and Table 4, the earliest node in the literature was an article published in *Foot & Ankle International* in 2004 entitled “The Lapidus Procedure: A Prospective Cohort Outcome Study.” Coetzee JC. et al. analyzed that the Lapidus procedure was a great alternative to treat moderate to severe HV deformities (35). In the same year, other authors published articles about additional surgical procedures for HV, such as Scarf and Chevron osteotomies (36). In 2005, Menz et al. suggested that as a simple and non-invasive screening tool, the Manchester scale could effectively reflect the degree of HV deformity determined by x-ray measurement of HV and intermetatarsal angles (37). In the same year, another of Menz et al.'s studies showed that HV has a adverse effect on gait patterns, which may lead to instability and fall risk in older adults, particularly when walking on irregular terrain. It has also gained scholars' attention to the health problems of the elderly with HV. In addition, LCS and GCS have higher scores in Robinson AHN's study. The authors thoroughly reviewed that while technically demanding, HV surgery had a high success rate in appropriately selected patients. However, few patients had a poor postoperative prognosis. Thus, randomized, controlled trials were needed to illustrate the factors that determine good outcomes (38) and a favorable validation result score was also necessary. In 2007, Coughliny MJ's article had a significant influence. Both LCS and GCS ranked second, with 178 and 244, respectively. This article stated that the severity of HV was not related to Achilles tendon or gastrocnemius tendon tightness, increased first-ray range of motion, bilaterality, or flat feet. The degree of preoperative angular deformity and increasing age were not associated with the range of motion of the first metatarsophalangeal joint (39). In 2007, Easley ME. et al. present the first American Orthopedic Foot and Ankle Society (AOFAS) guidelines for the operative treatment of HV. His paper provided information on how to select an appropriate surgical treatment based on the severity of HV. It guided clinical and scientific researchers to treat HV better (40). Hence, the research has gradually deepened. Researchers set out to comprehensively evaluate HV surgery in terms of recurrence and post-operative complications. In 2010, among all articles, Nix S's article had the highest LCS and GCS and was a classic document in the field of HV. HV was prevalent and became more common among aging women (6). From the historical nodes of HV, 70% of the nodes are in the period from 2004 to 2007, thereby indicating that researchers have a high degree of recognition for the literature quality of HV in this period. Various researchers focus on clinical studies of HV but note that the articles with higher scores are primarily reviews. Possibly because these high-quality papers are published by well-known research teams in high-impact journals, thereby enabling academics to rapidly and in greater detail understand the overall progress of HV and better guide clinical practice. Thus, studying this literature has led to a clearer understanding of the developmental trajectory of HV and the latest results.

**Table 4:** The historical direct citation network.

Rank	Tittle	Year	Type of study	LCS	GCS
1	The Lapidus Procedure: A Prospective Cohort Outcome Study.	2004	Clinical research	62	80
2	Scarf osteotomy for hallux valgus. A prospective clinical and pedobarographic study.	2004	Clinical research	80	109
3	Chevron osteotomy in hallux valgus—ten-year results of 112 cases.	2004	Clinical research	59	82
4	Prevalence of foot and ankle conditions in a multiethnic community sample of older adults.	2004	Clinical research	65	307
5	Radiographic validation of the manchester scale for the classification of hallux valgus deformity.	2005	Clinical research	69	98
6	Gait instability in older people with hallux valgus.	2005	Clinical research	60	87
7	Correlation of hallux valgus surgical outcome with AOFAS forefoot score and radiological parameters.	2005	Clinical research	61	82
8	Percutaneous distal metatarsal osteotomy for correction of hallux valgus.	2005	Clinical research	64	95
9	Modern concepts in the treatment of hallux valgus.	2005	Review	125	177
10	Hallux valgus: demographics, etiology, and radiographic assessment.	2007	Review	178	244
11	The shape of the lateral edge of the first metatarsal head as a risk factor for recurrence of hallux valgus.	2007	Clinical research	84	110
12	Radiographic results after percutaneous distal metatarsal osteotomy for correction of hallux valgus deformity.	2007	Clinical research	59	75
13	Hallux valgus and first ray mobility—a prospective study.	2007	Clinical research	91	124
14	Current concepts review: hallux valgus part II: operative treatment.	2007	Review	91	113
15	Prevalence and associations of hallux valgus in a primary care population.	2008	Clinical research	87	123
16	Postoperative incomplete reduction of the sesamoids as a risk factor for recurrence of hallux valgus.	2009	Clinical research	96	124
17	Factors associated with hallux valgus in a population-based study of older women and men: the mobilize Boston Study.	2010	Clinical research	75	105
18	Prevalence of hallux valgus in the general population: a systematic review and meta-analysis.	2010	Review	246	348
19	Impact of hallux valgus severity on general and foot-specific health-related quality of life.	2011	Clinical research	72	104
20	The pathogenesis of hallux valgus.	2011	Review	98	151

### Mutation detection by co–citation analysis

Co-citation analysis establishes a relationship between items based on how many times they have been cited together and has been demonstrated as a way to assist in identifying critical literature for cross-disciplinary ideas (41). Co-citation analysis helps researchers understand the past research priorities and predict the research direction of HV in the future. Figure 6B listed references with an outbreak duration of at least 2 years (2004–2021). The top 20 references with the most powerful citation bursts are depicted. The three articles with the strongest citation bursts were published in 2010, 2011, and 2017. First, among all studies, Nix et al.'s study had the highest strength (24.48). His article also had the highest LCS and GCS scores in the historical node. In addition, his article concluded the prevalence of HV in the general population through systematic review and meta-analysis (6). We also found that Menz et al.'s studies possessed a higher burst strength (14.55). He discovered that the general and foot-specific health-related quality of life decreased gradually as the severity of the HV deformity increased (42). In addition, Lee M's article had a high burst intensity (14.33). His study pointed out that with the increasing use of minimally invasive techniques for HV, the pain was significantly reduced in the first 6 weeks after percutaneous chevron/akin procedures compared to a traditional open scarf/akin osteotomies (43).

### Conceptual structural map

**Figure 7:**
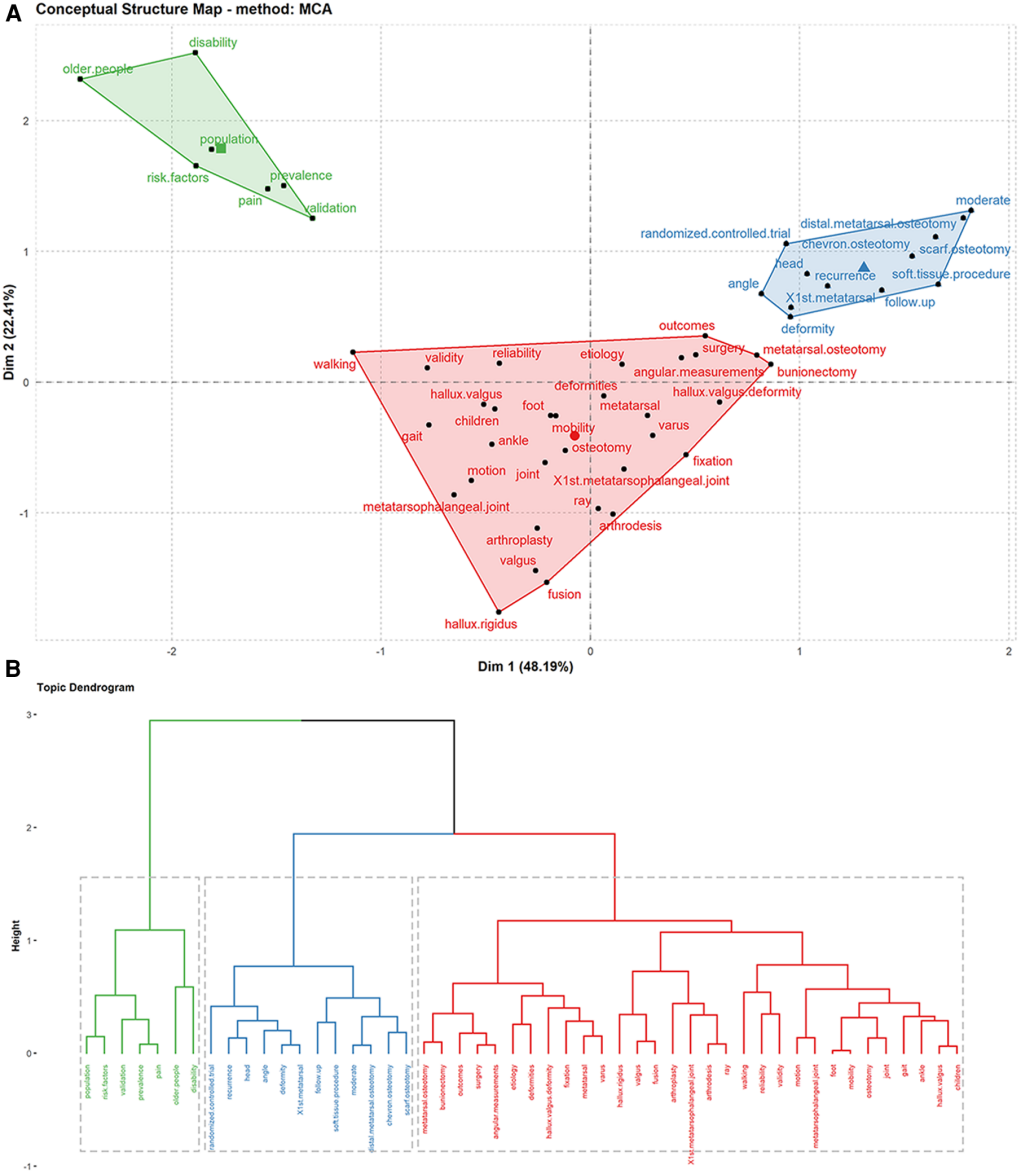
(**A**) multiple correspondence analysis of high-frequency keywords in HV. (**B**) Tree dendrogram of hierarchical cluster analysis of keywords in HV.

In bibliometrics, cluster and multiple correspondence analyses are commonly used methods for analyzing keywords (44, 45). As an independent tool, cluster analysis obtains the distribution of data, observes the characteristics of each cluster of data, and focuses on specific clusters for further analysis. Multiple correspondence analysis can reveal differences between categories of the same variable and correspondence between categories of different variables. Figures 7A,B showed several clustering results of correspondence analysis in the HV domain. Particularly, it can be divided into three categories.

The first cluster analysis (green topic): This category was primarily related to the risk factors and epidemiological investigation of HV (keywords: prevalence, pain, population, older people, and risk factors). The second cluster analysis (red topic): This category was primarily related to the diagnosis, examination, and surgical treatment of HV (Keywords: etiology, gait, ray, angular measurements, deformities, metatarsophalangeal joint, metatarsal osteotomy, bunionectomy, osteotomy, fusion, arthroplasty, and fixation). The third cluster analysis (blue topic): This category was primarily related to follow-up and recurrence after HV surgery. (Keywords: follow-up, recurrence, angle, deformity, and metatarsal).

### Analysis of research hotspots and trends

**Figure 8:**
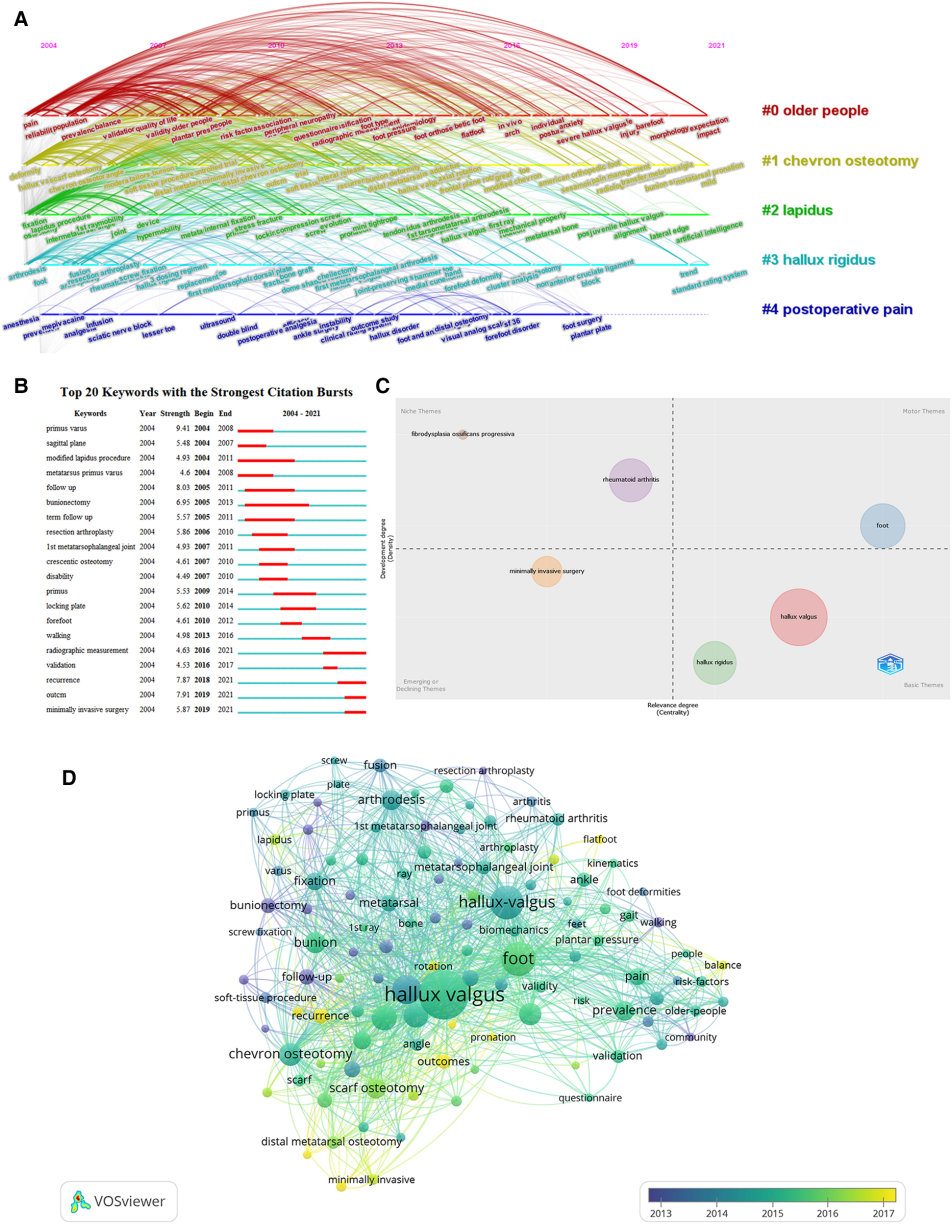
(**A**) timeline distribution of keywords cluster analysis. (**B**) The top 20 keywords with the most citation bursts. (**C**) Thematic map generated was displayed in four quadrants. (**D**) The overlay visualization map of the keywords co-occurrence analysis. The gradual development of color from blue to yellow indicated the evolution of hotspots.

Keywords are high-level summaries of research topics and content. The timeline view of keywords cluster analysis can outline the relationship between keywords and the period of important nodes. In Figure 8A, X and Y axes represent the year when the citation was published and the clustering number, respectively (14). Through analyzing the timeline view in the field of HV, “older people,” “chevron osteotomy,” “Lapidus,” and “hallux rigidus” have always been the hotspots of attention.

In contrast to common high-frequency words, burst keywords better reflect the dynamic deduction and development mechanisms of academic research. As shown in Figure 8B, the terms “radiographic measurement,” “recurrence,” “outcome,” and “minimally invasive surgery” were the latest popular keywords in the past 5 years. Developing trends and frontiers of research areas can be obtained by analyzing thematic maps. In Figure 8C, the thematic map generated by the R-Bibliometric software package was displayed in four quadrants, and the importance and centrality of the theme were represented by X and Y axes, respectively (46). The motor theme represented an important and well-developed theme, the niche theme represented a highly developed and isolated theme, the basic theme represented a basic area of research, and the emerging or declining themes emerge a new theme, that is, minimally invasive surgery. Co-occurrence analysis is a quantitative study of the phenomenon of co-occurrence to reveal the knowledge implied by the content associations and feature items of information. Keywords co-occurrence analysis not only identifies prevalent areas and research directions but is also an important indicator to monitor the development of scientific fields (15). An overlay visualization map of the co-occurrence analysis using VOSviewer for 114 keywords with more than 25 occurrences was illustrated in Figure 8D. All keywords were indicated by different colors, with blue keywords and yellow keywords indicating early and late appearance, respectively. The evolution of hotspots was indicated by the gradual development of color from blue to yellow.

Different bibliometric software emphasizes different aspects based on different algorithms. Thus, we comprehensively used the thematic map generated by the R-Bibliometric software package, the burst keywords of CiteSpace, and the overlay visualization map VOSviewer to identify six terms as the focus and the forefront of the field, which were “radiographic measurement,” “recurrence,” “outcome,” “rotation,” “pronation,” and “minimally invasive surgery” to comprehensively analyze the future trends in the field of HV.

## Discussion

### The general knowledge structure and major contributors (country, institution, author, and journal)

In this study, bibliometric and visual analysis was used to identify hotspots and collaborations across different countries, institutions, and authors to better understand the global research trends and hotspots of HV. Although the number of research papers published in HV slightly fluctuated from 2004 to 2021, there was a general tendency to increase. This indicates that an increasing number of researchers focus on the topic in HV.

Of the top 20 countries in terms of total publications, North America, Europe, and East Asia dominated the HV. With the rapid economic development of Asian countries, regional disparities are gradually narrowing. However, China and Turkey should also pay more attention to the quality of published papers. The United States had the most significant number of published articles and total citations and thus made an essential contribution to the field of HV.

As for institutions, La Trobe University in Australia and the Hospital for Special Surgery in the United States were not only the two with the most publications but also the highest collaboration networks. La Trobe University is known for its outstanding teaching and research capabilities and enjoys a global reputation. Meanwhile, the Hospital for Special Surgery is the earliest orthopedic hospital in the United States and has been ranked first in the list of the best orthopedic hospitals in the United States for 11 consecutive years. Not only is it the cradle of medical masters, but it is also a place of pilgrimage for orthopedic surgeons worldwide. The United States accounts for nearly half of the top 20 institutions in the field of HV, which can reflect the scientific research strength of American scientific research institutions. Thus, other scientific research institutions should also strengthen cooperation and exchanges.

Menz HB, Coughlin MJ, and Roddy E were the three most influential authors who might determine the focus and direction of the study. Menz HB, a foot and ankle specialist from La Trobe University in Australia, has been focusing on the research of footwear characteristics and foot problems of the elderly. Professor Coughlin MJ is an internationally renowned specialist in foot and ankle surgery who specializes in forefoot and hindfoot reconstructive surgery as well as total ankle reconstruction. Professor Roddy E's main contribution was to determine the population prevalence and factors associated with HV in the primary care population. They have been committed to research in the field of foot and ankle and made great contributions to its development. Therefore, we should place more emphasis on their work to keep up with the latest developments in this field.

Few researchers are familiar with all the important journals in their area. However, they often struggle to pick the appropriate journals to publish their research results. This conclusion may be obtained from journal metrics collected through bibliometric analysis. We also observed that most of the major papers on related studies in HV were published in professional journals such as *Foot & Ankle International*, *Journal of Foot & Ankle Surgery*, and *Foot and Ankle Surgery*. *Foot & Ankle International* has the highest number of publications, total citations, and H-index in this field. In addition, as a professional journal of foot and ankle surgery published in the United States and the official journal of the American Orthopedic Foot *&* Ankle Society (AOFAS), *Foot & Ankle International* has been emphasizing clinical research related to the foot and ankle joint. The journal primarily focuses on surgery, wound care, bone healing, pain management, diabetes, and sports medicine. *The Journal of Foot & Ankle Surgery* is the leading source for original, clinically focused articles on foot and ankle surgery and medical management. *Foot and Ankle Surgery* is the official journal of the European Foot and Ankle Society. The journal aims to advance the art and science of ankle and foot surgery, thus publishing peer-reviewed research articles. The reason for the greater attention paid to these journals may lie in the following: first, these journals have a high influence in the field of foot and ankle; second, the research directions covered are closely related to the clinical research related to HV; and third, researchers are more willing to promote their research results and opinions in well-known journals to improve their academic level and scientific research ability. The dual map overlay of journals makes the interdisciplinarity of the research possible, and following the trail of the map allows tracking the development of the scientific frontier (Figure 5E). The citing journals for all publications came from two main fields: (I) Medicine/Medical/Clinical and (II) neurology/sports/ophthalmology. Meanwhile, the reference journals (cited journals) originated from (I) Health/Nursing/Medicine; (II) Sports/Rehabilitation/Sport.

### The research topic, research hotspots, and research directions

Keywords and reference analysis are the essence of academic papers, which can reflect the current research hotspots and help researchers understand the evolution trend (47).

Cluster analysis helps to examine the conceptual structure of research. Multiple correspondence and cluster analyses showed three themes in the conceptual structure maps. The green topic was mainly related to the risk factors and epidemiological investigation of HV, the red topic was mainly related to diagnosis, examination, and surgical treatment of HV, and the blue topic was mainly related to follow-up and recurrence after HV surgery (Figure 7).

Through analyzing the timeline view of keywords in the field of HV (Figure 8A), “older people,” “chevron osteotomy,” “Lapidus,” and “hallux rigidus” have always been the hotspots of attention. Older people are at high risk for HV, and as the degree of HV deformity increases, the overall health and foot health of older adults gradually decline. Thus, managing foot function through proper care and control of the foot is necessary to prevent the appearance or progression of HV deformities (48, 49). The severity of the deformity determines the choice of the surgical plan. While mild-to-moderate HV is commonly treated with a distal operation like a Chevron osteotomy, more severe HV is generally treated with a proximal technique like Scarf osteotomy or Lapidus surgery. Chevron osteotomy is one of the most frequently used methods by foot and ankle surgeons worldwide, and the technique is constantly modified by the angle of the osteotomy with fixation (50). Meanwhile, as first metatarsal wedge fusion, Lapidus surgery is indicated primarily for patients with hypermobility of the first ray (51). Senga et al. believed that HV is a risk factor for HR (hallux rigidus). Although HV is one of the biological factors of HR, some researchers believe that current research has failed to provide a clear link (52). More pathophysiological studies of the relationship between HV and HR may be available in the future.

Through the analysis and evaluation of the research directions of the historical cited papers and the top 20 references with the most powerful citation bursts in the field of HV (Figures 6A,B), 16 (40%) publications were related to the changes and development of HV surgery, 8 (20%) were related to epidemiological research, 7 (17.5%) were related to radiological measurement, 3 (7.5%) were related to the efficacy comparison of the scoring system, and 3 (7.5%) were related to the analysis of the causes of postoperative recurrence. Moreover, 2 (5%) of the publications involved the impact of quality of life and 1 (2.5%) research was related to gait. Changes and developments in the surgery of HV have gained researchers' interest. Although there are more than 150 surgical types for HV, most have been eliminated (53). There are now more than 10 common procedures, which primarily include soft tissue surgery, arthroplasty, osteotomy, joint fusion, and joint replacement (54). In recent years, minimally invasive surgery has become increasingly important and is being assessed more scientifically (55). No operation is perfect and no one can solve all problems. Surgical plans need to be fully considered by experienced surgeons, including the severity of HV and the patient's needs (56). It is significant to note that surgical treatment of HV has greatly advanced from traditional open surgery to the present minimally invasive treatment.

### Future research trends

As HV research progresses, several emerging research directions are emerging as research topics of interest. After overlapping analysis of multiple bibliometric software (Figures 8B–D), the following seven keywords were identified as future research trends: “radiographic measurement,” “recurrence,” “outcome,” “rotation,” “pronation,” and “minimally invasive surgery.” Thus, focusing on progress in these research directions may lead to remarkable research results that will significantly advance the development and advancement of the field.

### Outcome, recurrence, rotation, and pronation

The surgical treatment of HV is primarily to relieve pain and correct deformity. However, postoperative recurrence is one of the most common complications after HV surgery. Recurrence is often caused by various factors, mostly related to preoperative evaluation, intraoperative technique, postoperative management, and patient reasons (57). Common risk factors for HV recurrence are severe HV deformity before the operation, metatarsal pronation, improper surgical selection or manipulation, and so on.

Preoperative HVA (hallux valgus angle) >40°, oversized IMA (intermetatarsal angle) can increase the probability of recurrence after HV surgery. Excessive adduction, elevation, and pronation of the metatarsals are also recognized risk factors for recurrence, particularly the coronal plane was unstable (58). HV is associated with the axial rotation of the first metatarsal, but failure to recognize and correct the malrotation of the first metatarsal may result in recurrent HV deformity. Wagner et al. concluded that identification of the anterior metatarsal rotation deformity is the key to achieving complete correction of the deformity and reducing the recurrence rate (59). Conti MS. et al. found that the recurrence rate was significantly lower in the group with reduced first metatarsal pronation than in the group with no change/increase in first metatarsal pronation (60). The traditional option of distal metatarsal osteotomy for severe HV deformities results in a high possibility of recurrence. Therefore, the ability to achieve good results and avoid recurrence also greatly depends on the degree of deformity correction and the surgeon's experience. Thus, individualized preoperative planning based on the patient's specific situation can effectively reduce the probability of recurrence (61). Future research may focus on the origins of HV disease and the causes of postoperative recurrence to develop better strategies for treating HV.

### Radiographic measurement

Radiographic measurement is the basic parameter and gold standard imaging modality to assess the severity of HV. Radiographs of foot weight-bearing are most commonly used in clinical practice to measure the HVA and IMA to assess the severity of HV and its degree of deformity (62). Radiographic measurement is essential for the surgical treatment of HV, as well as the correct positioning of the foot, the experience of the investigators, and so on, which can affect the accuracy of the measurements and may lead to incorrect decisions. A randomized controlled experiment found the radiographic measurement of the HV angle to be reliable by smartphone applications for both experienced and inexperienced investigators, and these tools improved the accuracy of the HV angle measurement and thus saved measurement time (63). With the rise of modern technology, future radiographic measurements of HV angle will be more precise and easier.

### Minimally invasive surgery

Percutaneous or minimally invasive procedures are becoming increasingly popular, and the results achieved in forefoot surgery are similar to those of the traditional open approach (10, 11, 64). Although there has been controversy about the effectiveness of minimally invasive techniques for HV, the clinical results are reliable in studies of minimally invasive surgery. Minimally invasive osteotomy has the advantages of less trauma, rapid recovery, and low complications. In a systematic review reporting a total of 1,762 patients (2,279 feet), investigators found little difference in outcomes between techniques. A meta-analysis reported that minimally invasive surgery was more effective than open surgery in the treatment of HV (65). In addition, better radiological and clinical outcomes were obtained in the minimally invasive group than in the open group (66). There may be some complications in minimally invasive surgery that are not present in open surgery, such as damage to soft tissue structures or skin burns that are not under direct visible control. However, minimally invasive surgery carries the risk of failure in patients with severe HV. Furthermore, minimally invasive techniques are the future trend of HV surgery treatment. With the continuous development and application of new technologies and improvement of surgical techniques, minimally invasive techniques for HV will be raised to another level, which will then provide better treatment for HV patients.

## Strengths and limitations

This is the first bibliometric article to analyze hotspots and trends in the field of HV from 2004 to 2021. Nevertheless, this research has certain limitations. First, an unavoidable limitation of bibliometrics is that it may lead to incomplete searches of the papers because of the restriction of search terms. Although it may affect the precision of the study section, it is unlikely to change the outcomes. Second, the WoSCC database is the most commonly used and comprehensive in bibliometrics. Although there are other types of databases, the WoSCC database is sufficient to reflect the field and dynamics of research considering the compatibility and recognition of bibliometric software. We finally obtained research data from this database. Third, only English articles and reviews were included in the data. Finally, there may be differences between bibliometric studies and real-world research.

## Conclusions

This article provides a comprehensive overview of the current status, hotspots, and trends in HV research from 2004 to 2021 through bibliometrics. The number of publications shows an overall increasing trend and the research of HV has a great research prospect. Thus, the United States has made an essential contribution to the field of HV. La Trobe University in Australia was the most productive institution. Menz HB and *Foot & Ankle International* were the most influential authors and the most popular journals among researchers, respectively. However, global research in HV is unevenly distributed. Thus, cooperation between countries and institutions should be strengthened. Meanwhile, “older people,” “chevron osteotomy,” “Lapidus,” and “hallux rigidus” have always been the hotspots of attention. Changes and developments in the surgery of HV have stimulated the interest of scholars. Future research trends are more focused on “radiographic measurement,” “recurrence,” “outcome,” “rotation,” “pronation,” and “minimally invasive surgery.” Thus, focusing on these subject directions can facilitate academic progress and provide the possibility of better treatments for HV. This study offers a valuable reference to help researchers, particularly new entrants, to better understand HV from a macroscopic perspective.

## Data Availability

The datasets presented in this study can be found in online repositories. The names of the repository/repositories and accession number(s) can be found in the article/Supplementary Material.
